# 

**Published:** 1881-02-20

**Authors:** 


### TABLE OF CONTENTS.

ORIGINAL AND SELECTED ARTICLES.

Conversations upon the Physical and Mental Hygiene of Girlhood, by T. S. Powell, M.D, Atlanta, 41. Hemorrhagic Fever successfully treated without quinine, by E. H. M. Parham, M.D, 48. Post-Partum Hemorrhage and its Treatment, by S. P. Sackett, M.D., N. Y., 50. Digestive Agents in Inflammatory Rheumatism, by S. V. Swentningen, M.D., Ind., 52. Notes on Post-Partum Hemorrhage, by F. E. Bodemann, M.D., Mich., 54. Prolapsus Ani successfully treated by Pypodermic Injections of Strychnia, by Leonard Weber, M.D., N. Y., 56.

ABSTRACTS AND GLEANINGS.

Treatment of Burns. 51. Iodoform in Gynæcology, 57. Eczema of the Genitals, 58. Vaccine Virus, 62. Treatment of Phthisis, 63. Pneumonia, 64. Dangers of Uterine manipulations and Operations, 65. Chloral in After-Pains, 66. Poisoned by Vegetables from Poisoned Soils, 67. Delirium Tremens; Apparent Death as a Result of Asphyxia, 68. Toothache; Injection of Ergotin in Acute Articular Rheumatism, 69. Diphtheria; Reprehensible practice; Chloral Cure for Toothache, 70.

SCIENTIFIC ITEMS.

Ozone; The Color of Ozone; Occurrence of Tails in Men, 71. The Physiology of walking; Strength of Insects, 72.

PRACTICAL NOTES AND FORMULÆ.

Diphtheria; A Vehicle for Salicylic Acid; Esmarck's Caustic Powder, 73. Bismuth Hair Dye; Salicylic Acid as a Foot Powder; Furnitute Polish; Hair Dye with one Preparation, 74. Peptized Milk as Food for Infants and Invalids; Effervescent Beverages; Nervine, 75. Bronchitis; Remedies for Chilblains; For Asthma; Treatment of Choleraic Diarrhœa by the Hypodermic Injection of Morphia, 76.

EDITORIAL AND MISCELLANEOUS.

Editorial Notices; Consultations, 77. Southern Medical College Hospital, 78. Medical Society of Pennsylvania; Book Notices, 79. Special Notices. 80.

